# The Sunday Effect: How Church Attendance is Related to Daily Pain and Affect in Older Adults with Osteoarthritis

**DOI:** 10.1093/geroni/igaa057.3385

**Published:** 2020-12-16

**Authors:** Shelley Condon, Katherine Cheesman, Dylan Smith, Patricia Parmelee

**Affiliations:** 1 The University of Alabama, Tuscaloosa, Alabama, United States; 2 Stony Brook University, Stony Brook, New York, New York, United States

## Abstract

Attending church is related to elevated mood (Law & Sbarra, 2009), greater social connectivity (Obst & Tham, 2009), and purpose in life (Robbins & Francis, 2000). More research is needed on how these relationships function among older adults, especially those living with chronic pain. The current study aimed to explore the effects of attending church on Sunday on the daily pain and affect of older adults with arthritis pain. Using a subset of 185 participants living in Alabama from the Everyday Quality of Life (EQUAL) project, the current study utilized multilevel modeling to examine (1) the main effects of church attendance and day of week (Sunday vs other) on daily pain and affect (positive and negative), and (2) the interaction [Sunday] effect of church attendance and day of week on those outcomes, controlling for sociodemographic variables (i.e., employment status, sex, race, and age). Preliminary covariate analyses revealed that church attendance was higher among participants who were unemployed, female, African American, and older. For the multilevel models predicting daily positive and negative affect, significant main effects were found for day of week and church attendance; however, the interaction effect was not significant. Interestingly, no main or interaction effects were found for the models predicting daily pain. Thus, while church attendance and day of week significantly predicted daily ratings of positive and negative affect, there was no support for the Sunday Effect on those outcomes. Implications and ideas for future research are discussed. (R01-AG041655, P. Parmelee & D. Smith, Co-PIs).

